# Structural Uncertainty in Onchocerciasis Transmission Models Influences the Estimation of Elimination Thresholds and Selection of Age Groups for Seromonitoring

**DOI:** 10.1093/infdis/jiz674

**Published:** 2020-03-16

**Authors:** Jonathan I D Hamley, Martin Walker, Luc E Coffeng, Philip Milton, Sake J de Vlas, Wilma A Stolk, Maria-Gloria Basáñez

**Affiliations:** 1 London Centre for Neglected Tropical Disease Research, Department of Infectious Disease Epidemiology, Imperial College London, London, UK; 2 Medical Research Council Centre for Global Infectious Disease Analysis, Department of Infectious Disease Epidemiology, Imperial College London, London, UK; 3 London Centre for Neglected Tropical Disease Research, Department of Pathobiology and Population Sciences, Royal Veterinary College, University of London, Hatfield, UK; 4 Department of Public Health, Erasmus University Medical Center, Rotterdam, The Netherlands

**Keywords:** onchocerciasis, Ov16 serology, receiver operating characteristic curve, age-dependent exposure, density dependence, elimination, threshold, positive predictive value, ivermectin, microfilarial prevalence

## Abstract

**Background:**

The World Health Organization recommends monitoring *Onchocerca volvulus* Ov16 serology in children aged <10 years for stopping mass ivermectin administration. Transmission models can help to identify the most informative age groups for serological monitoring and investigate the discriminatory power of serology-based elimination thresholds. Model predictions depend on assumed age-exposure patterns and transmission efficiency at low infection levels.

**Methods:**

The individual-based transmission model, EPIONCHO-IBM, was used to assess (1) the most informative age groups for serological monitoring using receiver operating characteristic curves for different elimination thresholds under various age-dependent exposure assumptions, including those of ONCHOSIM (another widely used model), and (2) the influence of within-human density-dependent parasite establishment (included in EPIONCHO-IBM but not ONCHOSIM) on positive predictive values for different serological thresholds.

**Results:**

When assuming EPIONCHO-IBM exposure patterns, children aged <10 years are the most informative for seromonitoring; when assuming ONCHOSIM exposure patterns, 5–14 year olds are the most informative (as published elsewhere). Omitting density-dependent parasite establishment results in more lenient seroprevalence thresholds, even for higher baseline infection prevalence and shorter treatment durations.

**Conclusions:**

Selecting appropriate seromonitoring age groups depends critically on age-dependent exposure patterns. The role of density dependence on elimination thresholds largely explains differing EPIONCHO-IBM and ONCHOSIM elimination predictions.

Onchocerciasis, also known as river blindness, is caused by infection with the filarial nematode *Onchocerca volvulus* and is targeted for elimination predominantly by mass drug administration (MDA) with ivermectin [1]. The World Health Organization (WHO) recommends monitoring transmission using serological tests to detect IgG4 antibodies against the Ov16 recombinant antigen [2]. Absent or low seropositivity in children (<0.1% has been proposed) indicates that transmission is either completely interrupted or suppressed to an extent that the parasite population is no longer sustainable and elimination will ensue. Key policy-relevant questions are: (1) which age group is most informative for making MDA stopping decisions based on serological monitoring? and (2) at what serological prevalence can treatment be safely stopped with minimal risk of resurgence, and is this threshold applicable in all epidemiological settings?

The current WHO recommendation of monitoring children aged <10 years [2] is based on the premise that individuals born after treatment began—into an environment of declining or absent onchocerciasis transmission—are unlikely to be exposed/infected (and therefore seropositive) if the intervention was effective. Older individuals, who have been exposed/infected in the past, are generally assumed to remain seropositive for life, although the dynamics of the antibody response to Ov16 are incompletely understood [3].

Using the onchocerciasis transmission model ONCHOSIM, Coffeng et al [4] found that, across a wide range of transmission and treatment settings, and for varying assumptions about seroconversion and seroreversion, 5–14 year olds were consistently more informative than 0–9 year olds in predicting ongoing transmission. However, the optimal age group for seromonitoring will also depend on model assumptions regarding age-related patterns of exposure to the blackfly (*Simulium*) vectors of *O. volvulus*. For example, if exposure of 0–9 year olds is relatively low—even in an endemic setting before intervention—then these individuals will provide little information on the transmission dynamics in the wider population and monitoring older individuals will be more useful. Alternatively, if exposure is relatively high in young children, even if it subsequently declines with age, monitoring of lower age groups might be more appropriate. There is evidence that age- (and sex-) dependent exposure to *O. volvulus* varies geographically [5], although the extent of this variation among transmission foci between and within countries is poorly documented (but see [6–8]).

In addition to the selection of appropriate age groups for serological monitoring, understanding the processes that drive parasite resilience under MDA, and acknowledging uncertainty associated with such processes, is critical for identifying serology-based elimination thresholds. A key difference between ONCHOSIM [4, 9, 10] and the EPIONCHO family of models [11–13] is the assumption of transmission intensity-dependent parasite establishment within humans. (Transmission intensity is measured as the number of infective L3 larvae potentially received by a person maximally exposed to blackfly bites in a year, the so-called annual transmission potential [14].) Density-dependent parasite establishment implies that as the transmission intensity (and an individual’s level of exposure) increases, the proportion of incoming parasites establishing in humans decreases (see [13] for a discussion). This assumption [12, 13, 15, 16], integrated into the EPIONCHO family of models but not considered by ONCHOSIM, permits capturing the relationship between microfilarial prevalence (the proportion of the population, typically aged ≥5 years, positive for *O. volvulus* skin microfilariae) and the annual biting rate (ABR, the number of vector bites/person/year), that has been recorded in African savannah settings [13]. Walker et al [12] present a comparison of the microfilarial prevalence versus ABR relationships predicted by the 2 models. Transmission intensity-dependent establishment of adult *O. volvulus* contributes to endemic stability and enhances parasite resilience under ivermectin MDA (increasing the parasite population’s ability to resurge from low levels), presumably leading to more stringent predicted serological thresholds indicative of elimination.

Using the recently developed individual-based transmission model EPIONCHO-IBM [13], we explore (1) the role of age-dependent exposure patterns in the selection of age groups for seromonitoring, and (2) the influence of transmission intensity-dependent parasite establishment within humans on serology-based elimination thresholds. We reconcile differences in predicted serological elimination thresholds between EPIONCHO-IBM and ONCHOSIM by modifying structural assumptions of the former to mimic those of the latter. We discuss our results in the context of optimal age-group selection for serological monitoring and the definition of seroprevalence thresholds indicative of elimination.

## METHODS

### EPIONCHO-IBM

EPIONCHO-IBM has been described by Hamley et al [13] as an analogue of the population-based EPIONCHO model [11, 12], tracking the number of adult *O. volvulus* worms of both sexes and microfilariae within individual (human) hosts. Host births and deaths are based on the typical demography of rural low-income communities in Africa and individuals are differentially exposed to blackfly bites, driving an overdispersed (aggregated) distribution of parasites among hosts. Treatment with ivermectin rapidly depletes skin microfilariae (the life stage infective to the blackfly vectors) and temporarily sterilizes female *O. volvulus* [17] such that, given for long enough and at high enough coverage, transmission can be interrupted, and the infection eliminated.

### Ov16 Antibody Dynamics

We assumed that approximately 80% of individuals in the population can mount a serological response [18], producing Ov16-specific antibodies (seroconverting) due to the presence of at least 1 adult worm [19]. Importantly, because these individuals are selected randomly and independently of the transmission intensity they experience, this assumption should not influence how informative different age groups are for predicting elimination. We assumed that there is no seroreversion (ie, that individuals do not become seronegative, or that if their antibody titres decline, these are still above the cutoff value for seropositivity; Vitaliano Cama, personal communication). This was not found to influence how informative different age groups were for predicting elimination in previous work [4]. The serological test is assumed to have 100% sensitivity and specificity, that is we model “true” seroprevalence, $p_{true}$. The seroprevalence observed through the lens of an imperfect diagnostic, $p_{obs}$, is calculated as,

$$\begin{array}{l} p_{obs}=\;p_{true}\cdot Sensitivity+(1-p_{true})\cdot (1-Specif\;icity) \end{array}$$ (1)

### Scenarios

We simulated infection trends during hypothetical MDA programs and recorded the seroprevalence in various age groups 1 year after the final round of MDA. Transmission dynamics were then simulated beyond the final round of MDA to test if elimination is achieved. With this information we can group simulations based on whether the seroprevalence is below a threshold 1 year after treatment and whether these simulations resulted in elimination.

**Figure 1.**
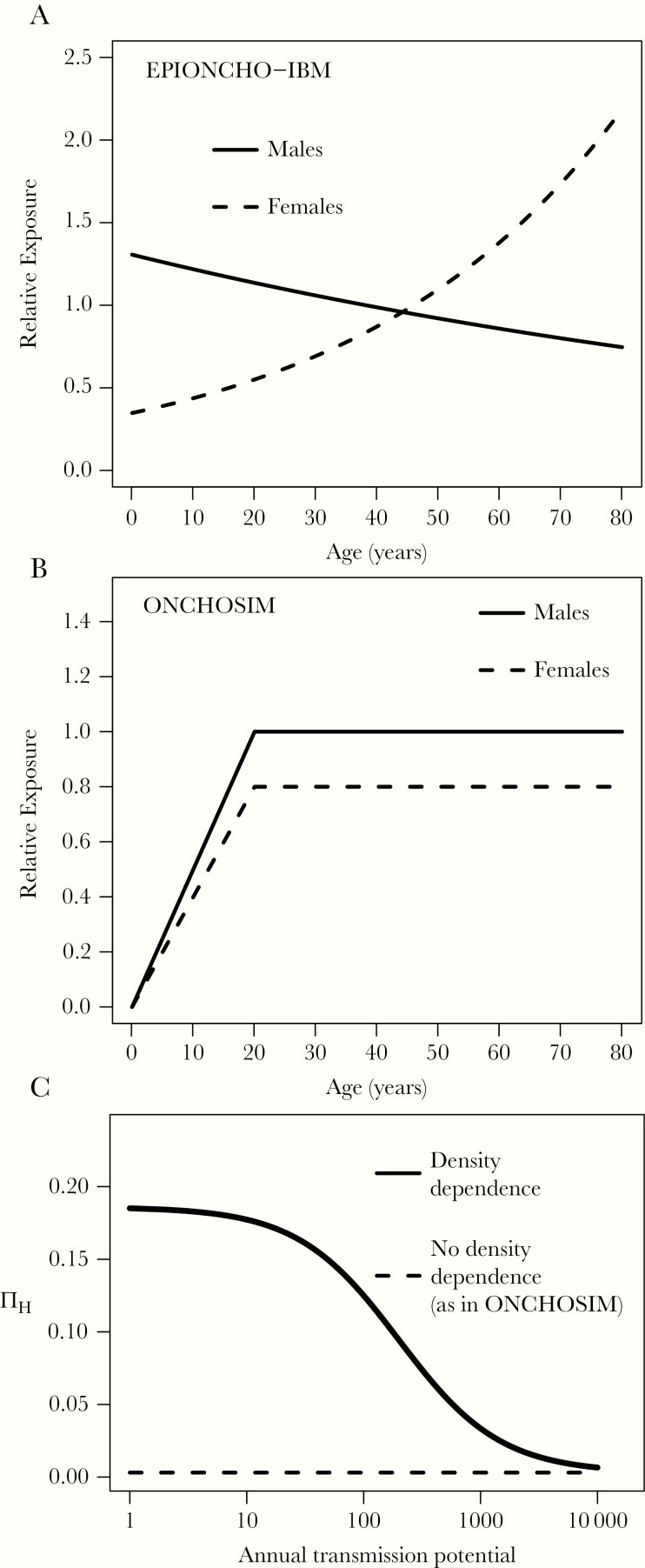
Age- and sex-dependent exposure functions, and proportion of parasites establishing within in humans in EPIONCHO-IBM and ONCHOSIM. The age- (in years) and sex-dependent patterns of relative exposure assumed in (*A*) EPIONCHO-IBM [13] and (*B*) ONCHOSIM [4, 10, 19]. *C*, the proportion of establishing parasites, ∏ _H_ (success ratio) as a function of the annual transmission potential (the number of L3 larvae/person/year, modelled as the annual biting rate multiplied by the mean number of L3 larvae in the fly population) in EPIONCHO-IBM (density dependence, monotonically decreasing solid line), and ONCHOSIM (constant, density-independent success ratio of 0.3%, horizontal dashed line). Note that in both EPIONCHO-IBM and ONCHOSIM, ∏ _H_ converges, independently, to the same success ratio value (0.3%) for high-transmission intensity settings [10, 13]. In panel C   we assume all individuals have the same exposure to fly bites, but account for exposure heterogeneity in the calculation of parasite establishment in the results of this paper (as in equation S2). See Hamley et al [13] for a discussion.

These simulations were conducted under various structural assumptions relating to age-dependent exposure and density-dependent parasite establishment within humans. In the following, we use the term “density dependence” to refer to transmission intensity (L3 larvae/person/year)-dependent parasite establishment within humans (both EPIONCHO-IBM and ONCHOSIM consider density [of skin microfilariae]-dependent parasite establishment within the simuliid vectors [10, 13]). Specifically, we investigated the following 4 scenarios: (1) EPIONCHO-IBM with no alterations (ie, age-dependent exposure as in [5] for savannah settings of northern Cameroon, and density-dependent establishment within humans as in [12, 13, 15, 16]); (2) EPIONCHO-IBM with age- and sex-dependent exposure to match that used in ONCHOSIM [4, 10] but with density dependence; (3) EPIONCHO-IBM with exposure as in [5] but without density-dependent parasite establishment (using a success ratio of 0.3%, as in ONCHOSIM, ie, the fraction of L3 larvae that develop into adults, which is independent of transmission intensity [4, 9, 10]); and (4) EPIONCHO-IBM with age- and sex-dependent exposure to match that used in ONCHOSIM and no density dependence. The forms of the age- and sex-dependent exposure functions and density-dependent adult *O. volvulus* establishment are shown in Figure 1 (see Supplementary Material for further details).

### Modelling Elimination

Because EPIONCHO-IBM is stochastic, the model is run 10 000 times for a given parameter set and the overall probability of elimination is the proportion of runs in 10 000 simulations that go to elimination. Elimination is assumed to occur when no parasites (in humans and flies) remain in the population 50 years after the last round of ivermectin MDA [7].

We assumed that a proportion of the population (nonadherent individuals) never take treatment (here 1% of the population), and that there was no treatment of children under the age of 5 years. Although the level of nonadherence is likely to vary between endemic communities (and may be higher than 1%), this should not qualitatively influence the differences in model predictions under the structural assumptions considered because nonadherence of individuals is assigned at birth and remains an attribute for life. Coverage was defined as the proportion of the total population that receive treatment at any given round (here 80%). Because some individuals are nonadherent or are <5 years old, therapeutic coverage never reaches 100% [10, 13].

### Selecting Age Groups for Seromonitoring

Following [4], we generated receiver operating characteristic (ROC) curves [20] for 6 age groups (0–4, 5–9, 0–9, 5–14, 10–14, and 15–19 years). A ROC curve for a given age group is calculated by plotting the true positive rate (TPR): the proportion of simulations in which the seroprevalence is below a threshold and results in elimination,

$$\begin{array}{l} TPR=\frac{true\;positives}{true\;positives+false\;negatives} \end{array}$$ (2)

against the true negative rate (TNR): the proportion of simulations in which the seroprevalence is above the threshold and does not result in elimination,

$$\begin{array}{l} TNR=\frac{true\;negatives}{true\;negatives+false\;positives} \end{array}$$ (3)

for a given range of serological thresholds. In Equation 3, for example, true negatives is the number of resurgence events in the modelled populations (ie, from 10 000 repeat simulations) in which the seroprevalence is greater than the serological threshold. (A full description of the terms in Equations 2 and 3 is given in Supplementary Table 1). The most informative age group for seromonitoring is defined as that which gives the largest area under the ROC curve [20].

### Identifying Serological Thresholds

Positive predictive values (PPVs) have been used to identify appropriate seroprevalence values at which treatment can be stopped (ie, elimination thresholds) [4, 21]. The PPV gives the probability of elimination when the seroprevalence (in the age group of interest), measured 1 year after the last MDA round, is below a given seroprevalence threshold,

$$\begin{array}{l} PPV=\frac{true\;positives}{true\;positives+false\;positives} \end{array}$$ (4)

Typically, at high seroprevalence, the PPV is equal to the overall elimination probability (ie, the proportion of all model runs in which elimination is achieved). The closer a PPV is to unity for a given seroprevalence threshold, the better the threshold is as a predictor of elimination. ROC plots indicate the age group giving the best balance between estimating ongoing transmission and elimination rather than the age group leading to the highest PPV.

**Figure 2.**
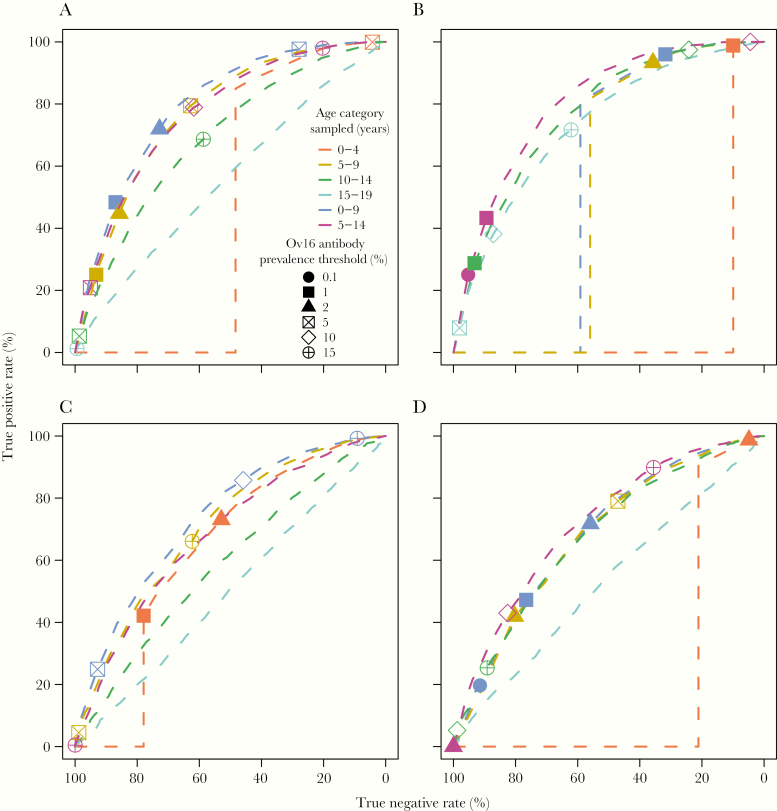
Receiver operating characteristic (ROC) curves for EPIONCHO-IBM with various exposure and density dependence assumptions. *A*, EPIONCHO-IBM (northern Cameroon) exposure and density-dependent adult worm establishment. *B*, ONCHOSIM exposure and density dependence. *C*, EPIONCHO-IBM exposure omitting density dependence. *D*, ONCHOSIM exposure omitting density dependence. Baseline microfilarial prevalence = 49% (*A*), 52% (*B*), 84% (*C*), and 80% (*D*), and years of treatment = 18 (*A*), 20 (*B*), 14 (*C*), and 13 (*D*). In all panels, treatment refers to annual ivermectin mass drug administration with 80% therapeutic coverage and 1% nonadherence. Each line assumes monitoring of a different age group. Note, results from the full ONCHOSIM model (with a similar probability of elimination, treatment coverage, and seroreversion assumption) can be found in the “Supplementary Material” of [4] in the ROC figure lattice for annual treatment, 80% coverage, and life-long seropositivity (page 10 of the document, specifically the panel for 5 years of treatment and a baseline community microfilarial load of 10 microfilariae/skin snip, giving an overall elimination probability of approximately 61%).

To facilitate vis à vis comparisons across structural assumptions, the ABR, leading to a baseline microfilarial prevalence and intensity of infection, and the number of years of annual ivermectin treatment simulated were altered for each scenario, such that the overall probability of elimination was the same for all scenarios. Therefore, we did not match all scenarios by baseline endemicity or treatment duration as in [4]; optimal comparisons were achieved when the overall probability of elimination was approximately 64%. Aligning microfilarial prevalence and/or treatment duration for the 2 density-dependence assumptions was found to give radically different elimination probabilities (frequently close to 0% when density dependence was assumed or to 100% when it was removed), decreasing the number of useable simulations for the generation of ROC curves. Thus, the baseline microfilarial prevalence and treatment duration were similar across exposure assumptions but differed notably between density-dependence assumptions (see the legend of Figure 2 for baseline prevalence values and years of treatment).

## RESULTS

### Age-Dependent Exposure and Selection of Age Groups for Seromonitoring

Changing assumed age-dependent exposure patterns (ie, switching the exposure pattern assumed for EPIONCHO-IBM to that in ONCHOSIM, depicted in Figure 1) influenced how informative the various age groups investigated were regarding ongoing (post-MDA) transmission. Generally, an inflection point (ie, indicating a change in the direction of the curvature) in the upper left corner of the ROC plot implies a more informative age group. Conversely, a curve closer to the 45-degree diagonal indicates a less informative age group.

When assuming the exposure in Figure 1A (northern Cameroon savannah [5]), 0–9 year olds were predicted to be more informative than 5–14 year olds (Figure 2A). The 5–9 year olds were predicted to be more informative than both 10–14 and 15–19 year olds, in contrast to the predictions when assuming ONCHOSIM exposure. When assuming ONCHOSIM exposure in EPIONCHO-IBM, 5–14 year olds were predicted to be the most informative age group (Figure 2B). By contrast, 0–9 year olds were 1 of the 3 least informative age groups (the others being 0–4 year olds and 5–9 year olds). The most informative age group for each exposure assumption was robust to the assumptions of density dependence (compare Figure 2A with Figure 2C and Figure 2B with Figure 2D). For the same overall probability of elimination (approximately 64%), model variants assuming density-dependent parasite establishment within humans (Figure 2A and Figure 2B) required lower baseline microfilarial prevalence (approximately 50%) and longer treatment duration (18–20 years) than model variants omitting density dependence (Figure 2C and Figure 2D), in which baseline prevalence was approximately 80% and treatment duration was 13–14 years.

### Threshold Estimation and Positive Predictive Values

**Figure 3.**
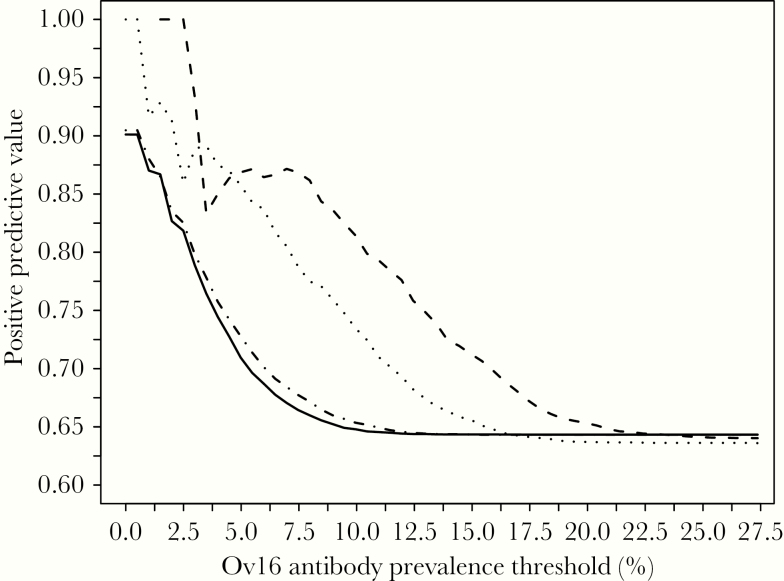
Positive predictive values versus Ov16 seroprevalence threshold (in percent) assuming serological monitoring of the most informative age groups as predicted by the ROC curves for each exposure and density dependence assumption. Solid line, EPIONCHO-IBM exposure, density dependence, and selection of 0–9 year olds for seromonitoring. Dot-dashed line, ONCHOSIM exposure, density dependence, and monitoring the 5–14 year olds. Dotted line, EPIONCHO-IBM exposure, no density dependence, and monitoring the 0–9 year olds. Dashed line, ONCHOSIM exposure, no density dependence, and monitoring the 5–14 year olds. Baseline microfilarial prevalence, treatment duration, coverage, and nonadherence as in Figure 2. Note, comparable results from the full ONCHOSIM model, with a similar probability of elimination, treatment coverage, and seroreversion assumption, can be found in the “Supplementary Material” of Coffeng et al [4] (page 23 of the document, in the PPV figure lattice for annual treatment, 80% coverage and life-long seropositivity, specifically the panel for 5 years of treatment and a baseline community microfilarial load of 10 microfilariae/skin snip, giving an overall elimination probability of approximately 61%).

The PPV gives the probability of elimination when the seroprevalence for the age group under consideration is below a given threshold. The 2 exposure assumptions gave similar PPV values when the monitored age group was based on the predictions of the ROC plots. However, for both exposure assumptions, when including density dependence, even a threshold seroprevalence of 0% did not achieve a PPV equal to 1 (Figure 3). The modelled scenarios regarding baseline microfilarial prevalence and treatment duration are the same as in the ROC analysis. For a given Ov16 seroprevalence threshold, the PPV values are higher for model variants that exclude density dependence, and go to 1 for low seroprevalence values (0.5% for EPIONCHO-IBM [northern Cameroon] exposure and 2.5% for ONCHOSIM exposure), whereas for model variants including density dependence, the maximum PPV value that can be achieved is 0.9 (90% elimination for seroprevalence thresholds of 0.5% for both exposure profiles). The PPV values converge to the overall approximately 64% probability of elimination as the value of the seroprevalence threshold increases (approximately 13% for model variants including density dependence and 20%–23% for models excluding it). The less smooth trajectory of the lines at lower seroprevalence values is due to the lower number of simulations that correspond to higher PPV values (a feature typical of this type of analysis [4]).

When increasing the monitored age group from 0–9 to 5–14 year olds and assuming EPIONCHO-IBM exposure and density dependence, a PPV of about 0.9 can be achieved with a wider range of seroprevalence thresholds (Supplementary Figure 1A, grey line). Note that based on the ROC curves, monitoring this older age group increases the probability of incorrectly predicting that there is ongoing transmission. In other words, when monitoring this older age group, the PPV is increased at the expense of an increased risk of continuing to treat a population for longer than necessary.

## Discussion

We have shown that the most informative age groups for seromonitoring will be influenced by age- (and sex-) dependent patterns of exposure. Identifying the most appropriate age groups for seromonitoring is a key question for policy makers designing recommendations and guidelines on robust approaches to measuring the elimination of onchocerciasis. EPIONCHO-IBM, with age- and sex-patterns of exposure as those inferred from microfilarial intensity age profiles from savannah settings in northern Cameroon [5] and density (transmission intensity) dependence in parasite establishment within humans, predicts that the 0–9 year olds would be the most informative age group (as stipulated in [2]). By contrast, altering age-dependent exposure patterns to reflect those used in ONCHOSIM resulted in 5–14 year olds being the most informative age group for serological monitoring, retrieving the predictions of the full ONCHOSIM model in terms of the most informative age group for serological monitoring, as published previously [4]. The selection of the most informative age groups according to exposure patterns was robust to assumptions on the operation or absence of density-dependent adult worm establishment, but the PPV values attained for a given seroprevalence threshold were highly sensitive to this assumption. When aligning the overall probability of elimination for the predictions with and without density dependence, the latter allowed a PPV of 1 when using the most informative age groups predicted by the ROC curves, unlike when density dependence was included. Importantly, without density-dependent adult worm establishment, a higher baseline microfilarial prevalence and fewer years of MDA were capable of generating a PPV of 1.

The selection of a seroprevalence threshold based on a desired PPV to decide when MDA can be stopped safely will be strongly influenced by the assumption of parasite establishment within humans being regulated by the intensity of *O. volvulus* transmission to which a community is exposed. Removing this regulation from EPIONCHO-IBM (to reflect ONCHOSIM’s assumption) resulted in the estimation of higher seroprevalence thresholds for a desired PPV value (when the PPV was above the overall probability of elimination). As an example, and for a PPV of 0.8, model variants including density dependence indicated that the seroprevalence threshold would approximately be 3%, whereas for model variants omitting density dependence this threshold was approximately 7% (Cameroon exposure) and approximately 11% (ONCHOSIM exposure). It must be emphasized that the magnitude of these values (or any others discussed here) corresponds to a hypothetical Ov16 test with perfect diagnostic performance (100% sensitivity and specificity). In practice, diagnostic performance is imperfect and the various Ov16 tests available (based on enzyme-linked immunosorbent assay or lateral flow assay/rapid diagnostic test platforms) vary in their sensitivity and specificity, with the desirable increase in the latter being obtained at the expense of the former [22]. Therefore, the 0.1% seroprevalence threshold proposed in [2] will have to be revised in the light of the results presented in [4], this work, and the characteristics of the test(s) that will be ultimately adopted by endemic countries to monitor their progress towards the onchocerciasis elimination goals [23].

To a large extent, the assumption of the absence of density-dependent adult worm establishment explains why ONCHOSIM is typically more optimistic on the prospects of elimination than the EPIONCHO family of models [12, 24, 25]. Hamley et al [13] discuss available evidence for the operation of this phenomenon in *O. volvulus* (based on [26]) and similarly in *Teladorsagia circumcincta,* in the sheep host [27]. In experimental filariasis, Babayan et al [28] showed that immune responses were stronger and adult worm recovery rates were lower as the number of infective L3 larvae of *Litomosoides sigmodontis* inoculated into BALB/c mice increased. The operation of density-dependent parasite establishment within humans and, importantly, the potential impact of treatment on this regulatory process and on the parasite’s reproductive biology are among outstanding uncertainties in the transmission dynamics and population biology of *O. volvulus* under chemotherapeutic intervention. These uncertainties have clear implications for the selection of seroprevalence thresholds indicative of elimination.

Coffeng et al [4] reported that monitoring of 0–9 year olds (as suggested in [2]), restricts the PPV to be below 1, even at a seroprevalence of 0. Our results suggest this finding is in part due to the age-dependent exposure patterns assumed in ONCHOSIM. In other words, monitoring of the 0–9 year olds may be appropriate if exposure is sufficiently high in younger children. However, when density dependence is assumed, monitoring of the most informative age group also imposes a constraint on maximizing the PPV. Thus, age-dependent exposure and density-dependent parasite establishment within humans may act together to constrain the PPV for a given seroprevalence threshold and monitored age group.

Seroreversion (immediately following the loss of the last parasite or as a gradual process of antibody titre decay following cessation of exposure; see [4] and [19] for a discussion about seroreversion), as well as imperfect test sensitivity and specificity [22, 23], would alter predictions of the PPV for a given threshold or alter the seroprevalence threshold necessary to achieve a given PPV. Although we did not explore the influence on our results of varying diagnostic performance and making different assumptions about seroreversion, it is unlikely that the qualitative patterns we report would be influenced by this assumption [4]. For example, although imperfect sensitivity would result in the prediction of lower seroprevalence thresholds for a given PPV, we would expect this to act similarly for the 2 exposure and density-dependence assumptions. There remains considerable uncertainty on the performance of Ov16 serology in Africa [29]. This must be better resolved to design suitable sampling protocols with the capacity to reliably measure serological thresholds [23].

In conclusion, geographical variation in age-dependent exposure may result in a fixed age group for sampling giving an inaccurate indication of ongoing transmission. This suggests data collection on age-related exposure will be a key step in developing more useful sampling regimes in near-elimination settings. This is difficult to measure directly, although it has been proposed that assays for antiblackfly saliva could be combined with assays for exposure to *O. volvulus* to investigate both exposure to vector bites and parasite antigens [13, 30]. A similar approach has been taken towards understanding heterogeneity in the transmission of *Leishmania infantum* among dogs using sandfly saliva assays [31]. Because density dependence and individual-level exposure heterogeneity (Equation S3 in the Supplementary Material) have been estimated simultaneously by fitting the model to preintervention ABR–microfilarial prevalence/intensity relationships, data collection on age-related exposure would also allow direct estimation of individual exposure heterogeneity, and thus reduce uncertainty in the estimation of density dependence [13]. Uncertainty in the processes regulating parasite establishment in humans largely explains the discrepancies in the predictions of the EPIONCHO family of models and ONCHOSIM. This represents a key area of outstanding uncertainty in the fundamental population biology of *O. volvulus,* which needs further research in order to offer greater precision on the likely magnitude of seroprevalence thresholds indicative of elimination.

## Supplementary Data

Supplementary materials are available at *The Journal of Infectious Diseases* online. Consisting of data provided by the authors to benefit the reader, the posted materials are not copyedited and are the sole responsibility of the authors, so questions or comments should be addressed to the corresponding author.

jiz674_suppl_Supplementary_Material

## Notes

***Author contributions*.** J. I. D. H., M. W., and M.–G. B. conceived the study. J. I. D. H. and P. M. developed the model. J. I. D. H. performed the analysis and prepared the figures. J. I. D. H., M. W., and M.–G. B. drafted the manuscript. All authors reviewed and approved the final version.

***Disclaimer*.** The views, opinions, assumptions, or any other information set out in this article should not be attributed to the Bill and Melinda Gates Foundation or any person connected with them. The funders had no role in the study design, data collection and analysis, decision to publish, or preparation of the manuscript.

***Financial support.*** This work was supported by the Bill and Melinda Gates Foundation via the NTD Modelling Consortium (grant number OPP1184344); UK Medical Research Council (MRC Doctoral Training Programme award to P. M.); MRC and the UK Department for International Development (DFID) Joint Centre Funding under the MRC/DFID Concordat agreement of the European Union European and Developing Countries Clinical Trials Partnership 2 (grant number MR/R015600/1 to J. I. D. H., P. M., and M.–G. B.); and the Dutch Research Council (grant number 016.Veni.178.023 to L. E. C.).

***Supplement sponsorship.*** The authors acknowledge funding of the NTD Modelling Consortium by the Bill and Melinda Gates Foundation (OPP1184344).

***Potential conflicts of interest.*** All authors: No reported conflicts of interest. All authors have submitted the ICMJE Form for Disclosure of Potential Conflicts of Interest. Conflicts that the editors consider relevant to the content of the manuscript have been disclosed.

Presented in part: Annual Meeting of the Coalition for Operational Research on Neglected Tropical Diseases, New Orleans, LA, 26–27 October 2018; and the European Congress on Tropical Medicine and International Health, Liverpool, UK, 16–20 September 2019.
