# Supplementary material for: Structural Uncertainty in Onchocerciasis Transmission Models Influences the Estimation of Elimination Thresholds and Selection of Age Groups for Seromonitoring
Source: J Infect Dis. 2020 Mar 16;221(Suppl 5):S510–8. doi: 10.1093/infdis/jiz674 (PMC7289547; doi:10.1093/infdis/jiz674)
Supplement: jiz674_suppl_Supplementary_Material [file jiz674_suppl_supplementary_material.docx]

**Supplementary Material**

**Structural uncertainty in onchocerciasis transmission models influences the estimation of elimination thresholds and selection of age groups for seromonitoring**

Jonathan I. D. Hamley^1,2*^, Martin Walker^1,3^, Luc E. Coffeng^4^, Philip Milton^1,2^, Sake J. de Vlas^4^, Wilma A. Stolk^4^, Maria-Gloria Basáñez^1,2^

1. London Centre for Neglected Tropical Disease Research (LCNTDR), Department of Infectious Disease Epidemiology, School of Public Health, Faculty of Medicine (St Mary’s campus), Imperial College London, Norfolk Place, London W2 1PG, UK.

2. MRC Centre for Global Infectious Disease Analysis, Department of Infectious Disease Epidemiology, School of Public Health, Faculty of Medicine (St Mary’s campus), Imperial College London, Norfolk Place, London W2 1PG, UK.

3. London Centre for Neglected Tropical Disease Research (LCNTDR), Department of Pathobiology and Population Sciences, Royal Veterinary College, University of London, Hatfield AL9 7TA, UK.

4. Department of Public Health, Erasmus MC, University Medical Center Rotterdam, P.O. Box 2040, 3000 CA Rotterdam, The Netherlands.

*Corresponding author Email: [jonathan.hamley11@imperial.ac.uk](mailto:jonathan.hamley11@imperial.ac.uk)

**Supplementary Methods**

**Preparation of Receiver Operator Characteristic (ROC) Curves and Calculation of Positive Predictive Values (PPV)**

The definitions provided in Supplementary Table 1 explain how to calculate the terms in equations (2) and (3) for the preparation of the ROC curves [1,2], and in equation (4) for the PPV values, which are found in the main text. A total of 10,000 repeat simulations were conducted for the modelled populations to calculate the *true positive rate (TPR)*, *true negative rate (TNR)*, and *positive predictive value (PPV)*.

**Supplementary Table 1. Definitions required to calculate Receiver Operator Characteristic (ROC) Curves and Positive Predictive Values (PPVs)**

| **Term** | **Definition (in 10,000 repeat simulations)** |
| --- | --- |
| TRUE NEGATIVES | The number of resurgence events in the modelled populations in which the seroprevalence is greater than the serological threshold |
| TRUE POSITIVES | The number of elimination events in the modelled populations in which the seroprevalence is less than or equal to the serological threshold |
| FALSE NEGATIVES | The number of elimination events in the modelled populations in which the seroprevalence is greater than the serological threshold |
| FALSE POSITIVES | The number of resurgence events in the modelled populations in which the seroprevalence is less than or equal to the serological threshold |

**Modelling Age- and Sex-Dependent Exposure and Density Dependence**

In EPIONCHO-IBM, age- and sex-dependent exposure is calculated following [3], using the parameterisation based on data from northern Cameroon savannah settings,

| $\Omega_{s\left( i \right)}=E_{s}\cdot\gamma_{s}\cdot{exp}^{\left[ -\alpha_{s}a_{\left( i \right)} \right]}$ | (S1) |
| --- | --- |

where $E_{s}$ is the sex-specific exposure to vector bites (calculated from the relative exposure of males versus females $Q={E_{M}}/{E_{F}}$, $\alpha_{s}$ is the sex-specific change in contact rate between vectors and hosts with increasing host age ($\alpha_{F}= -0.023 \mathrm{year}^{-1}, \alpha_{M}= 0.007 \mathrm{year}^{-1}, E_{M}= 1.08 , E_{F}= 0.9$) and $\gamma_{s}$ is a normalisation factor to ensure that the distribution of bites among age groups sums to 1. Each individual is assigned an exposure value at birth, $E_{(i)}$, resulting in total exposure being given by $\Omega_{T\left( i \right)}= \Omega_{s\left( i \right)}E_{(i)}$ [4].

Density-dependent establishment of adult *Onchocerca volvulus* in humans is given by,

| $\Pi_{H(i)}=\left[ \frac{{\delta_{H0}+\Omega_{T\left( i \right)}\delta}_{H\infty}c_{H}ATP}{1+{\Omega_{T\left( i \right)}c}_{H}ATP} \right]$ | (S2) |
| --- | --- |

where $\delta_{H0}$ is the proportion of incoming L3 larvae developing to the adult stage within the human host (per bite) when the annual transmission potential (*ATP*) tends to zero, $ATP\left( t \right)\to0$; $\delta_{H\infty}$ is the proportion of L3 larvae developing to the adult stage within the human host (per bite) when the *ATP* is very large, $ATP\left( t \right)\to\infty$, and $c_{H}$ is the, per L3 larva, severity of density dependence on parasite establishment within humans. The *ATP* is the annual number of infective, L3 larvae to which humans are exposed in a given community, and is modelled as the product of the annual biting rate (*ABR*, the number of bites/person/year), and the mean number of L3 larvae per (female) blackfly *Simulium* vector. Note that in equation (S2), the community-level ATP is distributed among humans according to their total individual exposure, $\Omega_{T\left( i \right).}$Parameter values are $\delta_{H0}$ = 0.186, $\delta_{H\infty}$ = 0.003, $c_{H}$ = 0.005 L3^–1^ year [4]. Density dependence is removed from EPIONCHO-IBM by setting $c_{H}$ = 0, and $\delta_{H0}$ = 0.0031 (the success ratio in ONCHOSIM [5]).

**Individual-Level Variation in Exposure to Blackfly Bites**

In both EPIONCHO-IBM and ONCHOSIM, each individual, in addition to a (relative) exposure based on their age and sex, is assigned an individual level exposure value which is drawn from a gamma distribution,

| $E_{(i)}\sim gamma(k_{E},\beta_{E})$ | (S3) |
| --- | --- |

where $k_{E}$ and $\beta_{E}$ are the shape and rate parameters, respectively, and $k_{E}$ = $\beta_{E}$, such that the mean individual-level exposure in the population is unity. Parameter $k_{E}$ has been estimated as 0.3 in EPIONCHO-IBM [4] and as 1–3.5 in ONCHOSIM [5]. Therefore, when removing density dependence from EPIONCHO-IBM, $k_{E}$ is set to 1. This alteration is partly justified by [4], as weaker exposure heterogeneity (i.e. a larger value of $k_{E}$) results in the estimation of weaker density dependence. Additionally, unpublished results with ONCHOSIM (W.A.S.) suggest that, in the absence of density-dependent parasite establishment in humans, strong overdispersion in the distribution of adult worms can be generated with values of $k_{E}$ ranging from 0.5 to 10. Therefore, to produce similar levels of parasite overdispersion between model variants including and omitting density dependence, the value of $k_{E}$ was increased in the latter.

**Supplementary Results**

Additional plots of the *positive predictive value (PPV)* vs. the Ov16 antibody prevalence threshold were obtained for several age groups explored using EPIONCHO-IBM. The left-hand plots (A) tend to show higher *PPV* values that decrease more slowly than those in (B), even when including density dependence, due to the assumption of higher levels of exposure in young children.

******

**Supplementary Figure 1. Positive predictive values (PPV) vs. Ov16 antibody prevalence threshold.** *A*, Using EPIONCHO-IBM with no alterations (age- and sex-dependent exposure patterns as in main text Figure 1A and density-dependent parasite establishment within humans as in the solid line of main text Figure 1C). *B*, EPIONCHO-IBM with ONCHOSIM exposure (main text Figure 1B) and density dependence. Baseline microfilarial prevalence = 49%–52%; treatment duration = 18–20 years; coverage = 80%; proportion of systematic non-adherers = 1%. The age groups investigated are 0–4 (dashed line), 5–9 (dotted line), 0–9 (solid black line), 10–14 (dot dash line), 5–14 (solid grey line) years old. Overall probability of elimination is approximately 64% as in the main text.

**Supplementary References**

1.Metz CE. Basic principles of ROC analysis. Sem Nuc Med **1978**; 8(4):283-298.

2. Coffeng LE, Stolk WA, Golden A, de Los Santos T, Domingo GJ, de Vlas SJ. Predictive value of Ov16 antibody prevalence in different sub-populations for elimination of African onchocerciasis. Am J Epidemiol **2019;** pii: kwz109 [Epub ahead of print].

3. Filipe JAN, Boussinesq M, Renz A, et al. Human infection patterns and heterogeneous exposure in river blindness. Proc Natl Acad Sci U S A **2005;** 102(42):15265-70.

4. Hamley JID, Milton P, Walker M, Basáñez MG. Modelling exposure heterogeneity and density dependence in onchocerciasis using a novel individual-based transmission model, EPIONCHO-IBM: implications for elimination and data needs. PLoS Negl Trop Dis (in press).

5. Coffeng LE, Stolk WA, Hoerauf A, et al. Elimination of African onchocerciasis: modeling the impact of increasing the frequency of ivermectin mass treatment. PLoS One **2014;** 9(12):e115886.
